# The effect and mechanism of Jiao-tai-wan in the treatment of diabetes mellitus with depression based on network pharmacology and experimental analysis

**DOI:** 10.1186/s10020-021-00414-z

**Published:** 2021-12-07

**Authors:** Yueheng Tang, Hao Su, Hongzhan Wang, Fuer Lu, Kexin Nie, Zhi Wang, Wenya Huang, Hui Dong

**Affiliations:** 1 Institute of Integrated Traditional Chinese and Western Medicine, Tongji Hospital, Tongji Medical College, Huazhong University of Science and Technology, Wuhan, 430030 Hubei China; 2 Department of Integrated Traditional Chinese and Western Medicine, Tongji Hospital, Tongji Medical College, Huazhong University of Science and Technology, Wuhan, 430030 Hubei China

**Keywords:** Jiao-tai-wan, Diabetes mellitus, Depression, Chronic restraint stress, Network pharmacology, Mechanism

## Abstract

**Background:**

The incidence of diabetes mellitus (DM) and depression is increasing year by year around the world, bringing a serious burden to patients and their families. Jiao-tai-wan (JTW), a well-known traditional Chinese medicine (TCM), has been approved to have hypoglycemic and antidepressant effects, respectively, but whether JTW has such dual effects and its potential mechanisms is still unknown. This study is to evaluate the dual therapeutic effects of JTW on chronic restraint stress (CRS)-induced DM combined with depression mice, and to explore the underlying mechanisms through network pharmacology.

**Methods:**

CRS was used on db/db mice for 21 days to induce depression-like behaviors, so as to obtain the DM combined with depression mouse model. Mice were treated with 0.9% saline (0.1 ml/10 g), JTW (3.2 mg/kg) and Fluoxetine (2.0 mg/kg), respectively. The effect of JTW was accessed by measuring fasting blood glucose (FBG) levels, conducting behavioral tests and observing histopathological change. The ELISA assay was used to evaluate the levels of inflammatory cytokines and the UHPLC-MS/MS method was used to determine the depression-related neurotransmitters levels in serum. The mechanism exploration of JTW against DM and depression were performed via a network pharmacological method.

**Results:**

The results of blood glucose measurement showed that JTW has a therapeutic effect on db/db mice. Behavioral tests and the levels of depression-related neurotransmitters proved that JTW can effectively ameliorate depression-like symptoms in mice induced by CRS. In addition, JTW can also improve the inflammatory state and reduce the number of apoptotic cells in the hippocampus. According to network pharmacology, 28 active compounds and 484 corresponding targets of JTW, 1407 DM targets and 1842 depression targets were collected by screening the databases, and a total of 117 targets were obtained after taking the intersection. JTW plays a role in reducing blood glucose level and antidepressant mainly through active compounds such as quercetin, styrene, cinnamic acid, ethyl cinnamate, (R)-Canadine, palmatine and berberine, etc., the key targets of its therapeutic effect include INS, AKT1, IL-6, VEGF-A, TNF and so on, mainly involved in HIF-1 signal pathway, pathways in cancer, Hepatitis B, TNF signal pathway, PI3K-Akt signal pathway and MAPK signaling pathway, etc.

**Conclusion:**

Our experimental study showed that JTW has hypoglycemic and antidepressant effects. The possible mechanism was explored by network pharmacology, reflecting the characteristics of multi-component, multi-target and multi-pathway, which provides a theoretical basis for the experimental research and clinical application of JTW in the future.

**Supplementary Information:**

The online version contains supplementary material available at 10.1186/s10020-021-00414-z.

## Introduction

Diabetes mellitus (DM) is a metabolic disease characterized by hyperglycemia with a rapidly increasing prevalence. According to the International Diabetes Federation (IDF), about 463 million adults worldwide suffered from DM in 2019, and the number is expected to reach 700 million in 2045 (IDF 2019). China has the largest number of DM patients in the world, with an incidence of 11.2% (Li et al. 2020). Not only DM itself is seriously harmful to human health, but also the complications will bring a heavy burden to the family and society. Depression, one of the most prevalent disorders of mental health that limits psychosocial functioning and diminishes quality of life, is a common psychological complication of diabetes (Malhi and Mann 2018). A meta-analysis showed that 14.5% of patients with type 2 diabetes mellitus (T2DM) were complicated with depression (Wang et al. 2019), and depression is twice as common in people with DM as in the general population (Moulton et al. 2015). Long-term depression not only affects patients' compliance with treatment, but also causes neuroendocrine dysfunction and increases blood glucose level, and poor blood glucose control will aggravate patients' depression. Therefore, it is urgent to strengthen early identification and give corresponding psychological or drug intervention.

At present, the commonly used antidepressants are selective serotonin reuptake inhibitors (SSRIs) and serotonin and noradrenaline reuptake inhibitors (SNRIs). According to reports, psychopharmacological treatment with SSRIs medications has a moderate-to-large effect on depression with lesser effects on glycemic control (Sartorius 2018). Another cohort study showed that antidepressant use is associated with the risk of diabetes onset in a time- and dose-dependent manner, the adjusted hazard ratio is 3.95 for long-term high-dose antidepressant use (Miidera et al. 2020). Hence in-depth exploration of the pathogenesis and development of drugs with the dual effects of improving of DM and depression is imminent.

Traditional Chinese medicine (TCM) is a holistic medical system which uses experience-based therapies such as acupuncture and herbal medicine (Xu et al. 2013). Jiao-tai-wan (JTW), originated from the *Han Shi Yi Tong* in the Ming Dynasty, is composed of two herbal medicines: Huanglian (HL, *Rhizoma Coptidis*) and Rougui (RG, *Cinnamon*), and it has been used to treat insomnia since ancient times. With the development of science and technology and the verification of experiments, the therapeutic effect of JTW on DM has been discovered (Zou et al. 2017; Chen et al. 2013, 2017). Since insomnia and depression are closely related, in recent years, more and more studies have confirmed that JTW have obvious antidepressant effects in addition to reducing blood glucose level and improving sleep quality. Experimental research results showed that JTW can significantly alleviate the depressive-like behavior of mice (Zhe et al. 2017; Xiang et al. 2020). Therefore, JTW may have dual effects in the treatment of DM and depression. However, whether JTW has such dual effects and its related mechanisms are not yet clear, especially the molecular target mechanisms of its effective components, which needs to be further explored.

Network pharmacology is a new discipline based on the theories of systems biology, bioinformatics and classical pharmacology. The new method for analyzing the targets and mechanisms of drug treatment of diseases from multiple angles provides the possibility to reveal the mechanism of TCM, which is booming in recent years and has been widely used in the field of TCM (Chen et al. 2018). Network pharmacology, as a useful tool, can help us to further understand the role of drugs and how we can improve drug discovery for complex diseases (Hopkins 2007).

**Fig. 1:**
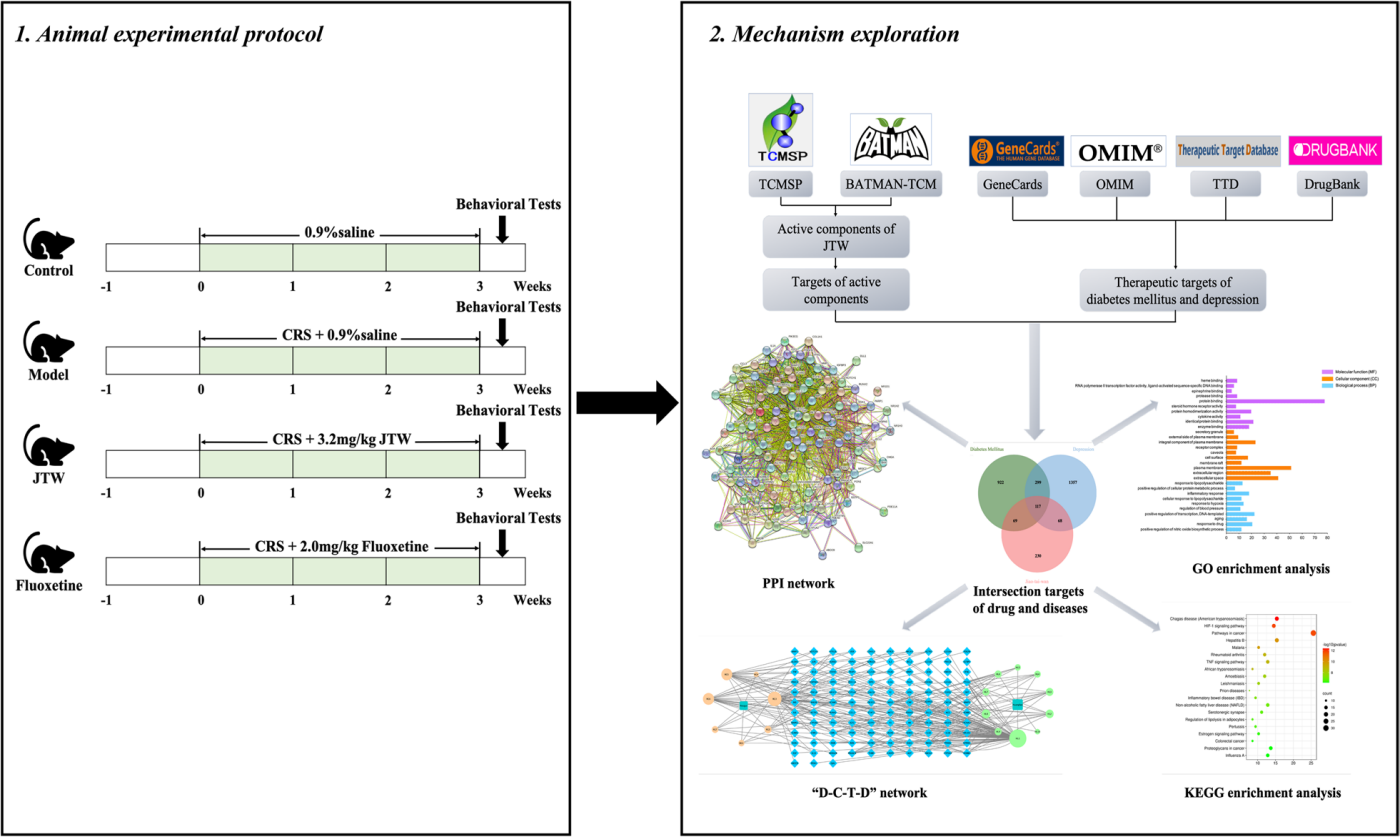
Workflow of the study of JTW on diabetes mellitus and depression and mechanism exploration based on network pharmacology analysis. *TCMSP* Traditional Chinese Medicine Systems Pharmacology Database and Analysis Platform; *BATMAN-TCM* Bioinformatics Analysis Tool for Molecular Mechanism of Traditional Chinese Medicine; *OMIM* Online Mendelian Inheritance in Man; *TTD* Therapeutic Target Database; *JTW* Jiao-tai-wan; *PPI* protein–protein interaction; *D-C-T-D* Drug-Compounds-Targets-Disease; *GO* Gene Ontology; *KEGG* Kyoto Encyclopedia of Genes and Genomes

Therefore, in our present study, we established a mouse model of DM combined with depression induced by chronic restraint stress (CRS) to evaluate the dual therapeutic effects of JTW, and the underlying mechanisms were explored through network pharmacology. The study procedure is shown in Fig. 1.

## Materials and methods

### Preparation of JTW and Fluoxetine

The ratio of HL and RG in JTW is 10:1 (w/w). Both HL and RG herbal concentrate-granules were purchased from China Resources Sanjiu Medical and Pharmaceutical Co., Ltd (Guangdong, China). As 1 g of HL and RG granules is as efficacious as 6 g of HL and 3 g of RG decoction pieces, the two granules were mixed at a ratio of 5:1 (w/w) and dissolved in ddH_2_O. Fluoxetine, which is the most commonly used drug for depression, was chosen as the positive control. The Fluoxetine pills were obtained from PATHEON FRANCE Co., Ltd. and were ground into powder and mixed with ddH_2_O sufficiently before gavage.

### Animals

The animal experiment was overseen and approved by the Animal Ethics Committee of Tongji Medical College, Huazhong University of Science and Technology. Twenty-four db/db mice (male, aged 7 weeks) were purchased from GemPharmatech Co., Ltd (Jiangsu, China) and maintained in the experimental animal center of Tongji Hospital (SPF-grade) with environmental conditions of 20 ± 2 °C, 60 ± 5% relative humidity and 12 h dark/light cycle.

### Preparation of animal model and treatment

Since db/db mice are spontaneous T2DM model mice, chronic restraint stress (CRS) was used to induce depression-like behaviors to obtain the DM combined with depression mouse model. After 1-week of acclimatization, the mice were randomly divided into four groups (six mice in each group): (1) Normal control group (Control), (2) Chronic restraint stress group (Model), (3) Jiao-tai-wan treatment group (JTW), (4) Fluoxetine treatment group (Fluoxetine). Mice in the JTW group and Fluoxetine group were gavaged daily with Jiao-tai-wan (3.2 mg/kg) and Fluoxetine (2.0 mg/kg), respectively, while the control and model groups received vehicle (0.9% saline). Drug treatment lasted from the 1st day of CRS until the end of this study. During the study, body weight of each mouse was measured every 3 days and fasting blood glucose (FBG) was measured once a week. At the last day of CRS, a glucose tolerance test (GTT) was performed on each mouse, all mice were fasted overnight and gavaged with glucose (2 g/kg), then the tail vein blood glucose was measured at 0, 30, 60, 90 and 120 min after gavage by glucose strip (ACCU-CHEK Performa, Roche).

Except for the control group, mice were exposed to CRS by placing in 100 ml plastic tubes with a few holes to keep air flow for 4 h per day for 21 days, they were able to move their forelimbs and head, but not their body. Mice in the control group were fasted at the same time without restraint, when the restraint procedure finished, the mice were returned to their cages immediately. Behavioral tests were then performed.

### Behavioral tests

#### Forced swimming test (FST)

The FST was based on a previously described with slight modifications (Slattery and Cryan 2012). Mice were placed separately in a clear glass cylinder (30 cm in height and 18 cm in diameter) filled with 20 cm water (24 ± 2 °C). The first 2 min of the entire 6 min test was used for adaptation and the duration of immobility was recorded in the next 4 min. A mouse was considered immobility when it stayed moveless and floating, or moved only to keep it head above the water. The FST was carried out in a quiet environment, with no obvious changes in light. After each trial, the mouse was removed from the water and returned to the cage, and the water in the cylinder was changed.

#### Open field test (OFT)

The OFT was based on a previously described (Shieh and Yang 2020). The OFT apparatus consisted of a 50 × 50 × 35 cm square box, divided into 16 equal-size squares. The central area of the apparatus was defined as a central 25 cm × 25 cm square, and the rest was the peripheral area. Each mouse was placed in a corner of the box, and the total distance travelled were recorded during the 5 min test. The OFT was performed in a quiet environment, with no obvious changes in light. 75% ethanol was used to clean the apparatus to remove odors after each trial.

#### Tail suspension test (TST)

The TST was also based on a previously described (Iyer et al. 2019). The rear 1/3 of the tail of each mouse was affixed with adhesive tape and suspended about 40 cm above the floor for 6 min. The immobility time was measured for the last 4 min. Immobility was defined as a lack of active escape movements and maintaining a vertical posture during suspension.

### Measurement of neurotransmitters and inflammatory cytokines

After all behavioral tests completed, all mice were anaesthetized with 1% pentobarbital (65 μl/10 g) to collect blood samples. Serum was obtained from the blood samples by centrifugation at 12,000 rpm for 15 min and stored at − 80 °C. Serum tumor necrosis factor-α (TNF-α), interleukin-6 (IL-6) and high-sensitivity C reactive protein (hs-CRP) levels were determined using the ELISA kits (ABclonal Technology, Boster Biological Technology Co., Ltd), according to the manufacturer’s instructions. Depression-related neurotransmitters such as Noradrenaline (NE), serotonin (5-HT), and dopamine (DA) levels were measured using UHPLC-MS/MS method.

### Hippocampus histopathology

The brain of each mouse was quickly isolated after blood collection for histological analysis. The hippocampus from mice in each group was obtained and fixed in 10% formalin, embedded in paraffin, and sliced into sections (4 μm thick). The sections were stained with Terminal Deoxynucleotidyl Transferase-Mediated dUTP Nick End-Labeling (TUNEL) according to the standard protocol. The hippocampus of each section was observed and photographed using an Olympus BX51 system (Olympus, Japan).

### Network pharmacology

#### Screening of active compounds and targets of JTW

The active compounds and corresponding targets of JTW were screened using the Traditional Chinese Medicine Systems Pharmacology Database and Analysis Platform (TCMSP, https://tcmspw.com/tcmsp.php) (Ru et al. 2014). The oral bioavailability (OB) and drug-likeness (DL) contained in this database are important indicators for evaluating ADME attributes, which are often used as the key factors to screen active compounds of drugs (OB ≥ 30% and DL ≥ 0.18)(Lee et al. 2018; Tao et al. 2019). We also chose those two indicators as the screening criteria in our study. The Bioinformatics Analysis Tool for Molecular Mechanism of Traditional Chinese Medicine (BATMAN-TCM, http://bionet.ncpsb.org.cn/batman-tcm/) was also used to screen the active compounds and potential targets (Liu et al. 2016). According to the parameter information given by the BATMAN-TCM database, the Score cutoff ≥ 30 and *p* value ≤ 0.05 were considered as indicators when screening the active compounds of JTW.

In addition, we also searched the relevant literature on the compounds of JTW and completed the data collection to avoid omitting some important active compounds and corresponding target information due to the setting of screening criteria. Meanwhile, target genes of these active compounds were predicted by the above databases and corrected by the Uniprot database (https://www.uniprot.org/).

#### Collection of targets of DM and depression

The targets of DM and depression were also obtained by searching the databases. With the key words including “depression”, “depressive”, “depressive disorder”, “depressive illness”, “diabetes” and “diabetes mellitus”, targets related to DM and depression were founded in the GeneCards (https://www.genecards.org/, version 5.1), the Online Mendelian Inheritance in Man (OMIM, https://omim.org/), the DrugBank (https://go.drugbank.com/, version 5.1.8) and the Therapeutic Target Database (TTD, (Stelzer et al. 2016; Wang et al. 2020; Wishart et al. 2018; Hamosh et al. 2005).

#### Construction and analysis of the “D-C-T-D” network

The targets of active compounds and diseases were collected and sorted out to obtain the intersection targets of JTW, DM and depression. According to these intersection targets, the Venn diagram was obtained by using jvenn platform [(Bardou et al. 2014). Afterwards, we integrated the information of the intersection targets and input them into Cytoscape software (https://cytoscape.org/, version 3.8.2] to construct a “D-C-T-D” network and complete the subsequent analysis. Cytoscape is a software platform for the large-scale integration of molecular interaction network data, and can integrate these networks with annotations, gene expression profiles and other state data (Shannon et al. 2003).

#### Construction of the protein–protein interaction (PPI) network

The intersection targets of JTW, DM and depression were uploaded to the STRING database (https://www.string-db.org/, version 11.0) to construct the PPI network of protein–protein interaction and the key targets were screened and analyzed subsequently. We set the scoring condition to > 0.90, and the selected target proteins were limited to *Homo sapiens*. In the PPI network, the edges represent protein–protein associations, and the more lines, the greater the correlation (Szklarczyk et al. 2021).

#### Gene Ontology (GO) and Kyoto Encyclopedia of Genes and Genomes (KEGG) enrichment analyses

The database for annotation, visualization and integrated discovery (DAVID, https://david.ncifcrf.gov/, version 6.8) was used to carry out GO enrichment analysis and KEGG enrichment analysis on the intersection targets, which is a web-based online bioinformatics resource that can be used to explain the functions of large lists of genes/proteins (Jiao et al. 2012). The GO enrichment analysis includes three different biological aspects: biological process (BP), molecular function (MF) and cellular component (CC) (Ashburner et al. 2000). KEGG is a knowledge base for systematic analysis of gene functions (Kanehisa and Goto 2000).

### Statistical analysis

Statistical analyses were performed by the GraphPad Prism 8 software and all the data are presented as the mean ± standard deviation (S.D.). Significant differences among the groups were evaluated with a one-way analysis of variance (ANOVA) and Dunnett’s t-test, and p < 0.05 was considered as statistically significant.

## Results

### JTW has a therapeutic effect on db/db mice

**Fig. 2:**
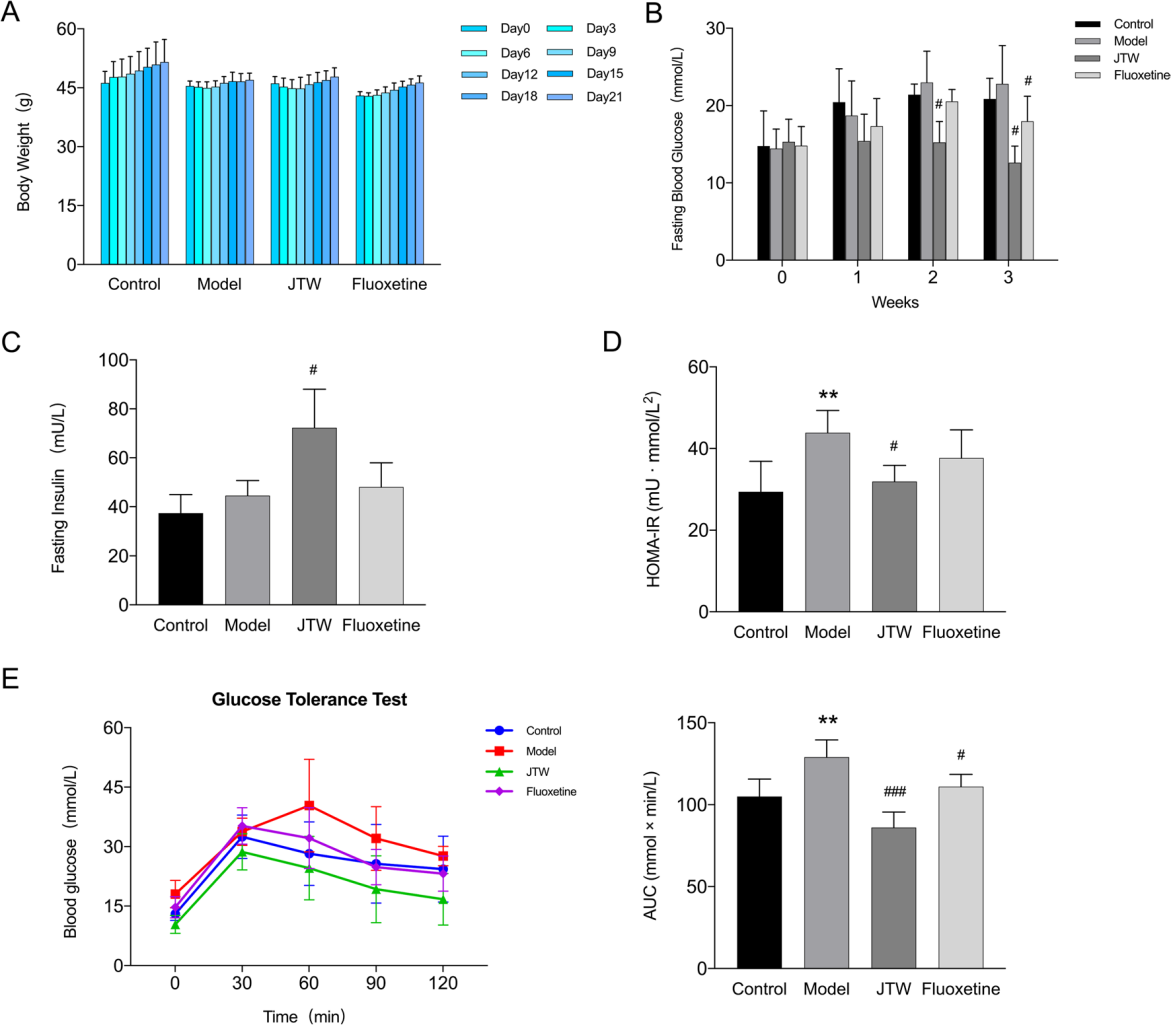
**A** Body weight of each mouse was recorded every 3 days (n = 5–6). **B** Fasting blood glucose (FBG) of each mouse was determined once a week (n = 5–6). **C** Fasting insulin (FINS) of each mouse was determined at the ending of study (n = 5–6). **D** HOMA-IR index was calculated according to standard formula: HOMA-IR = FBG (mmol) × FINS (mU/l)/22.5(n = 5–6). **E** For glucose tolerance test (GTT), all mice were fasted overnight and gavaged with glucose (2 g/kg), then the tail vein blood glucose was measured at 0, 30, 60, 90 and 120 min after gavage; the bar graph represents average area under the curve (n = 5–6). All data are presented as means ± SD. Compared to control group, *p < 0.05, **p < 0.01; Compared to model group, ^#^p < 0.05, ^##^p < 0.01, ^###^p < 0.001

In our study, the well-accepted and spontaneous T2DM model, db/db mice, were used to explore the effect of JTW in vivo. We recorded the body weight and FBG levels to evaluate the therapeutic effects of JTW, it can be seen that JTW had a trend of weight loss although it was not statistically significant (Fig. 2A). In terms of FBG levels, there was no difference in FBG between different groups at the beginning, while FBG in JTW and Fluoxetine groups were significantly decreased compared to Model group with the drug intervention (Fig. 2B). In addition, the serum samples were used to examine fasting insulin (FINS) level and calculated the homeostasis model assessment insulin resistance (HOMA-IR) index. According to following formula: HOMA-IR = FBG (mmol) × fasting insulin (mU/l)/22.5, we found that JTW obviously improved fasting insulin level and decreased HOMA-IR index (Fig. 2C, D). What’s more, we also conducted GTT to access glucose metabolism state, the changes were also similar with above parameters (Fig. 2E). All above indicated that JTW has a therapeutic effect on diabetic mice.

### JTW ameliorates depression-like behavior induced by CRS

**Fig. 3:**
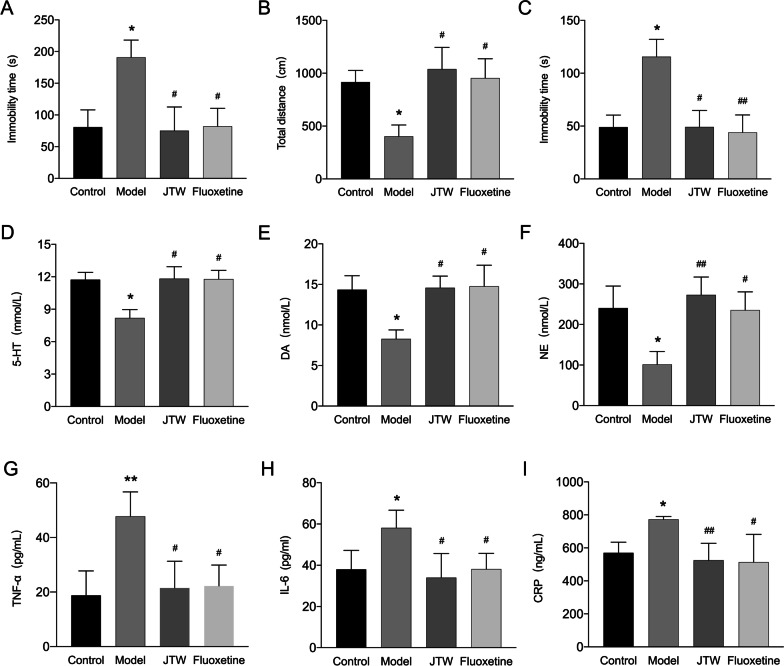
**A** The forced swimming test (FST) were conducted after chronic restraint stress (CRS) treatment for 21 days, the immobility time of each mouse was recorded in the last 4 min (n = 5–6). **B** The open field test (OFT) were performed after chronic CRS treatment for 21 days, the total distance travelled of each mouse was recorded during the 5 min test (n = 5–6). **C** The tail suspension test (TST) were conducted after CRS treatment for 21 days, the immobility time of each mouse was recorded in the last 4 min (n = 5–6). **D**, **E** Serum depression-related neurotransmitters such as Noradrenaline (NE), serotonin (5-HT), and dopamine (DA) levels were detected at the end of the experiment (n = 5–6). **G**, **H**, **I** Serum tumor necrosis factor-α (TNF-α), interleukin-6 (IL-6) and high-sensitivity C reactive protein (hs-CRP) levels were determined at the end of the experiment (n = 5–6). All data are presented as means ± SD. Compared to control group, *p < 0.05, **p < 0.01; Compared to model group, ^#^p < 0.05, ^##^p < 0.01

Mice received JTW or Fluoxetine 1 h before the restraint stress for 21 days to access the effect of JTW on CRS-induced depressive symptoms. During the FST for testing levels of depression, the immobility time was increased significantly in the model group compared to the control group, which was attenuated by both JTW and Fluoxetine (Fig. 3A). The results of the TST were similar to the FST, the model group showed obviously higher immobility time compared to the control group, while JTW and Fluoxetine groups showed significant reductions in immobility time compared to the model group (Fig. 3C). In OFT as shown in Fig. 3B, treatment with JTW or Fluoxetine significantly increased the total distance travelled when compared to the model group. These results suggest that JTW could alleviate depression-like behavior in mice induced by CRS.

### Effects of JTW on depression-related neurotransmitters

In addition to behavioral tests, we also measured the depression-related neurotransmitters such as 5-HT, DA and NE levels in the serum. As shown in Fig. 3D–F, exposure to chronic restraint stress obviously suppressed serum 5-HT, DA and NE levels, which could be significantly improved following Fluoxetine and JTW treatment.

### Effects of JTW on serum inflammatory biomarkers

Due to the close relationship between inflammation, DM and depression, we determined the inflammatory biomarkers levels using the ELISA kits. The pro-inflammation cytokines TNF-α, IL-6 and hs-CRP levels increased significantly after 21 days of CRS in the model group mice. Treatment with JTW and Fluoxetine decreased the cytokines in the serum, suggesting that JTW may play an effective role in attenuating inflammation (Fig. 3G–I).

### Effects of JTW on CRS-induced apoptosis in hippocampus

**Fig. 4:**
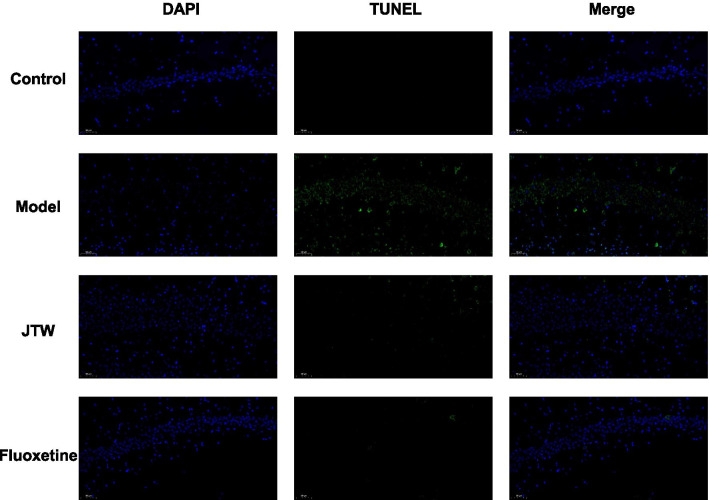
TUNEL staining was used to detect the apoptosis in the hippocampus of each mouse in different groups, the green dots indicate the TUNEL-positive apoptotic cell

The apoptosis level in the hippocampus of each mouse in different groups was detected via TUNEL staining. It can be seen that the number of TUNEL-positive cells in the hippocampus of mice in the model group was obviously higher than that of the control group, while almost no TUNEL-positive cells was observed in the hippocampus of mice in JTW and Fluoxetine groups (Fig. 4). The results revealed that CRS intervention markedly increase the apoptotic rate of hippocampal neurons, which can be significantly reversed by JTW and Fluoxetine treatment.

### Screening of active compounds and targets of JTW

After searching the TCMSP database and the BATMAN-TCM database with the criteria of OB ≥ 30%, DL ≥ 0.18, and Score cutoff ≥ 30, combined with the results of related literature(Kin et al. 2013; Ma et al. 2018; Chae et al. 2019), a total of 28 active compounds of JTW were initially obtained, namely 14 of HL and 14 of RG. The relevant information of these 28 active compounds can be found in the Additional file 1: Table S1. What’s more, 526 corresponding targets were collected from the two databases, including 179 of HL and 347 of RG. After standardizing and unifying target names and deleting duplicate targets, a total of 484 drug targets were obtained.

### Collection of therapeutic targets of DM and depression

After simplifying the results of each database and screening and removing duplicate targets, a total of 1842 targets of depression were obtained with the key words such as “depression”, “depressive”, “depressive disorder” and “depressive illness”. Similarly, 1407 targets of DM were obtained by using “diabetes” and “diabetes mellitus” as the key words to search in the databases.

### Screening of the intersection targets and constructing the D-C-T-D network

**Fig. 5:**
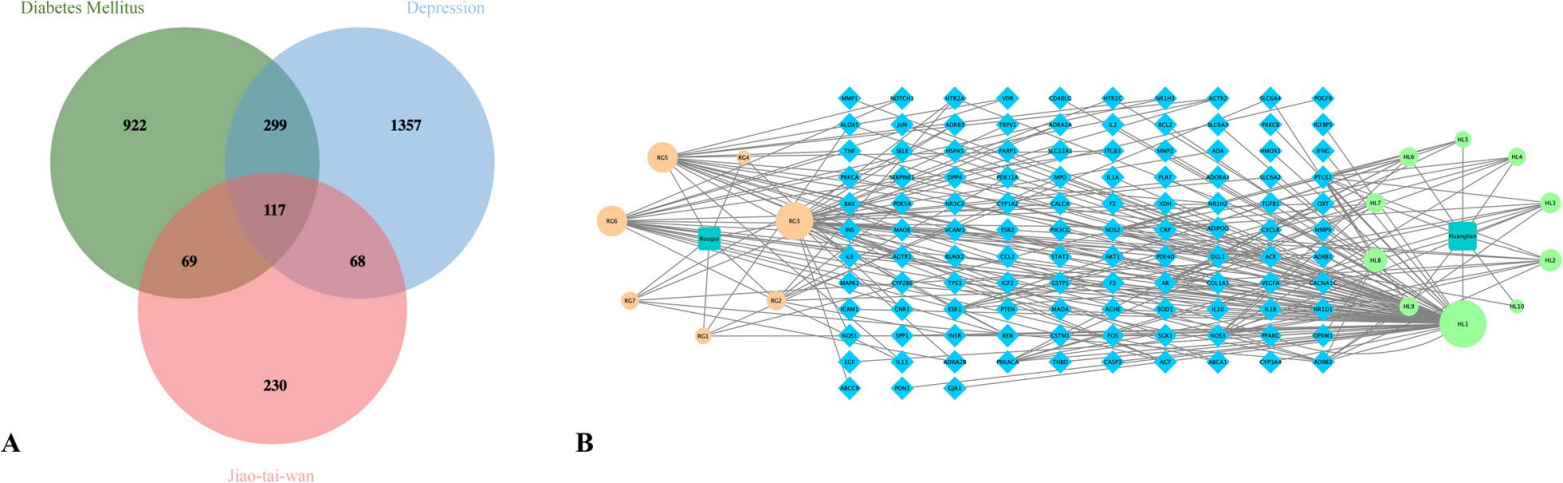
**A** The 117 intersection targets of Jiao-tai-wan, diabetes mellitus and depression. **B** The Drug-Compounds-Targets-Disease (D-C-T-D) network

Through taking the intersection of 484 drug targets, 1842 depression targets and 1407 DM targets, we obtained a total of 117 targets (Fig. 5A), which correspond to 17 active compounds (Table 1), suggesting that JTW probably performs the therapeutic effect via modulating the 117 genes. Subsequently, we input the information of the intersection targets into Cytoscape software for analysis in order to explore the possible mechanism of the therapeutic effect of JTW. Figure 5B shows the D-C-T-D network constructed by the Cytoscape, which fully reveals the multi-component and multi-target mechanism of JTW in the treatment of DM and depression. In addition, the analyzer tool that comes with the software was used to analyze the active compounds. The results showed that the active compounds with the highest number of targets included quercetin, styrene, cinnamic acid, ethyl-cinnamate, (R)-Canadine, palmatine, berberine, etc., indicating that the above compounds may be the main components of JTW in treating DM and depression.

**Table 1:** Seventeen active compounds of Jiao-tai-wan and their corresponding oral bioavailability (OB) and drug-likeness (DL)

Mol ID	Molecule name	OB (%)	DL
MOL000098	Quercetin	46.43	0.28
MOL000105	Protocatechuic acid	25.37	0.04
MOL000475	Anethole	32.49	0.03
MOL000704	Styrene	29.55	0.01
MOL000785	Palmatine	64.6	0.65
MOL000991	Cinnamaldehyde	31.99	0.02
MOL001454	Berberine	36.86	0.78
MOL001458	Coptisine	30.67	0.86
MOL002295	Cinnamic acid	19.68	0.03
MOL002668	Worenine	45.83	0.87
MOL002834	Ethylcinnamate	20.54	0.04
MOL002894	Berberrubine	35.74	0.73
MOL002897	Epiberberine	43.09	0.78
MOL002903	(R)-Canadine	55.37	0.77
MOL002904	Berlambine	36.68	0.82
MOL002907	Corchoroside A_qt	104.95	0.78
MOL003526	Cinnamyl acetate	21.15	0.04

### Construction and analysis of the PPI Network

**Fig. 6:**
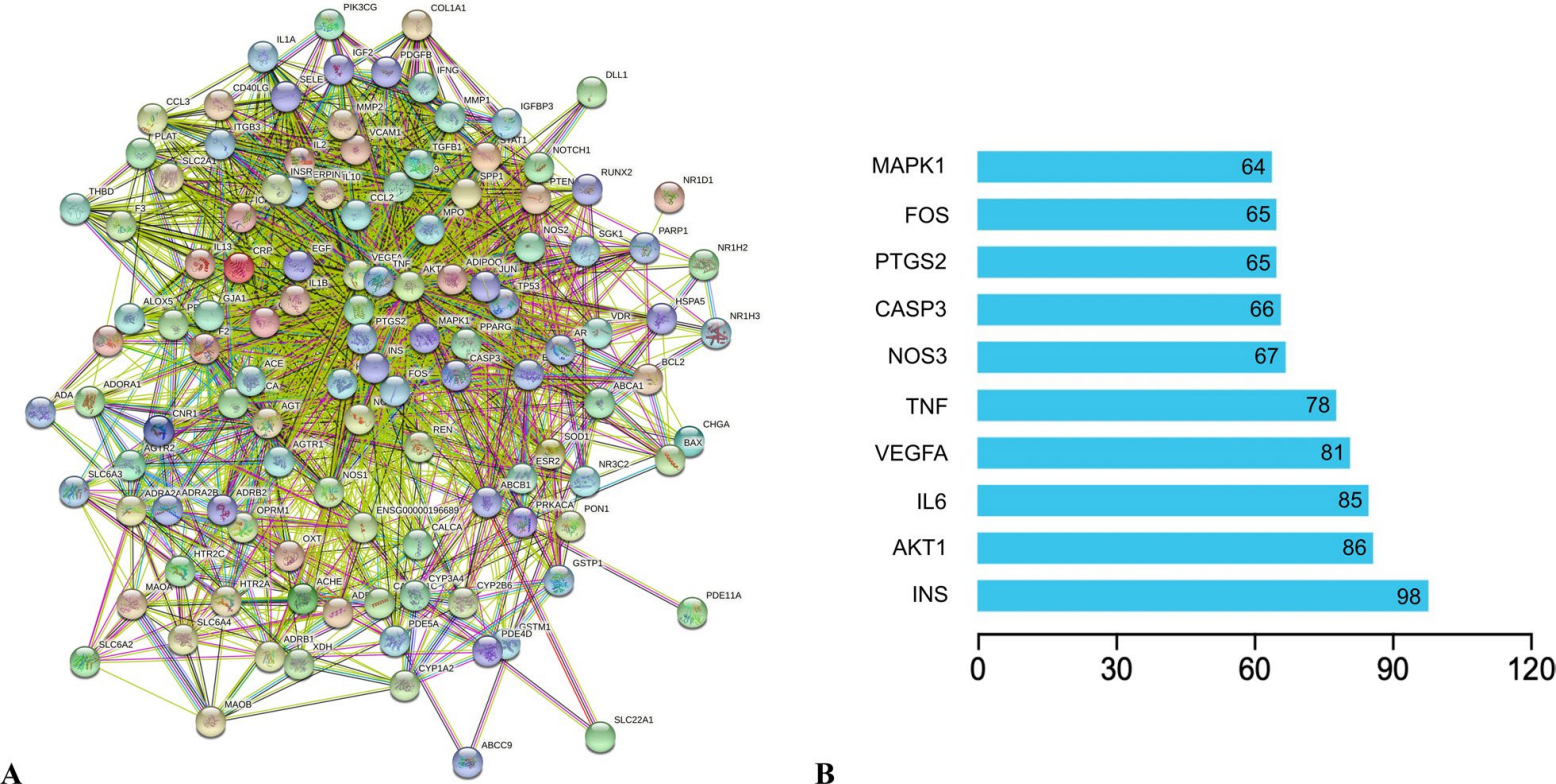
**A** The protein–protein interaction (PPI) network of 117 intersection targets. **B** The bar plot of the top 10 targets in the PPI network

117 intersection targets were uploaded to the STRING database to obtain the PPI network. There are 117 nodes and 1907 edges in the PPI network, and we screened out the top 10 targets after analyzing each node, which had a node degree greater than 64 (Fig. 6A, B). Therefore, we speculate that JTW may play a role in the treatment of DM and depression through the key targets such as INS, AKT1, IL-6, VEGF-A, TNF and so on.

### GO enrichment analysis

**Fig. 7:**
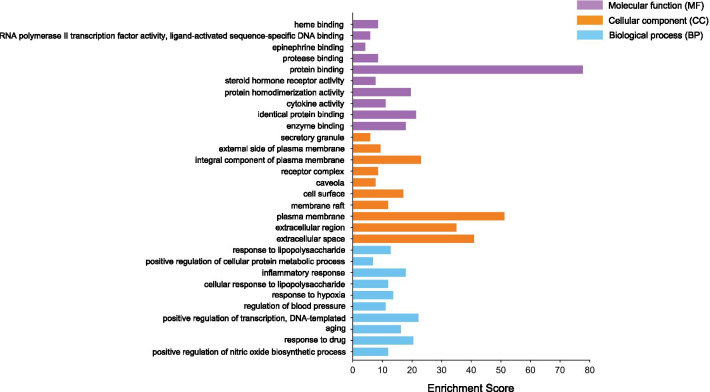
The top 10 biological process (BP), molecular function (MF) and cellular component (CC) in GO analysis of 117 intersection targets

The DAVID database was used to perform GO enrichment analysis of 117 intersection targets to explore the relationship between these targets and diseases, including three aspects of BP, MF and CC. The top ten results were output after ranking according to the *p* value from small to large (Fig. 7). It can be seen that the occurrence of DM and depression involves many biological processes, and JTW can achieve the purpose of treatment by regulating multiple biological processes. Additional file 2: Fig. S1 shows the bubble chart results of the top 20 in the GO enrichment analysis.

### KEGG enrichment analysis

**Fig. 8:**
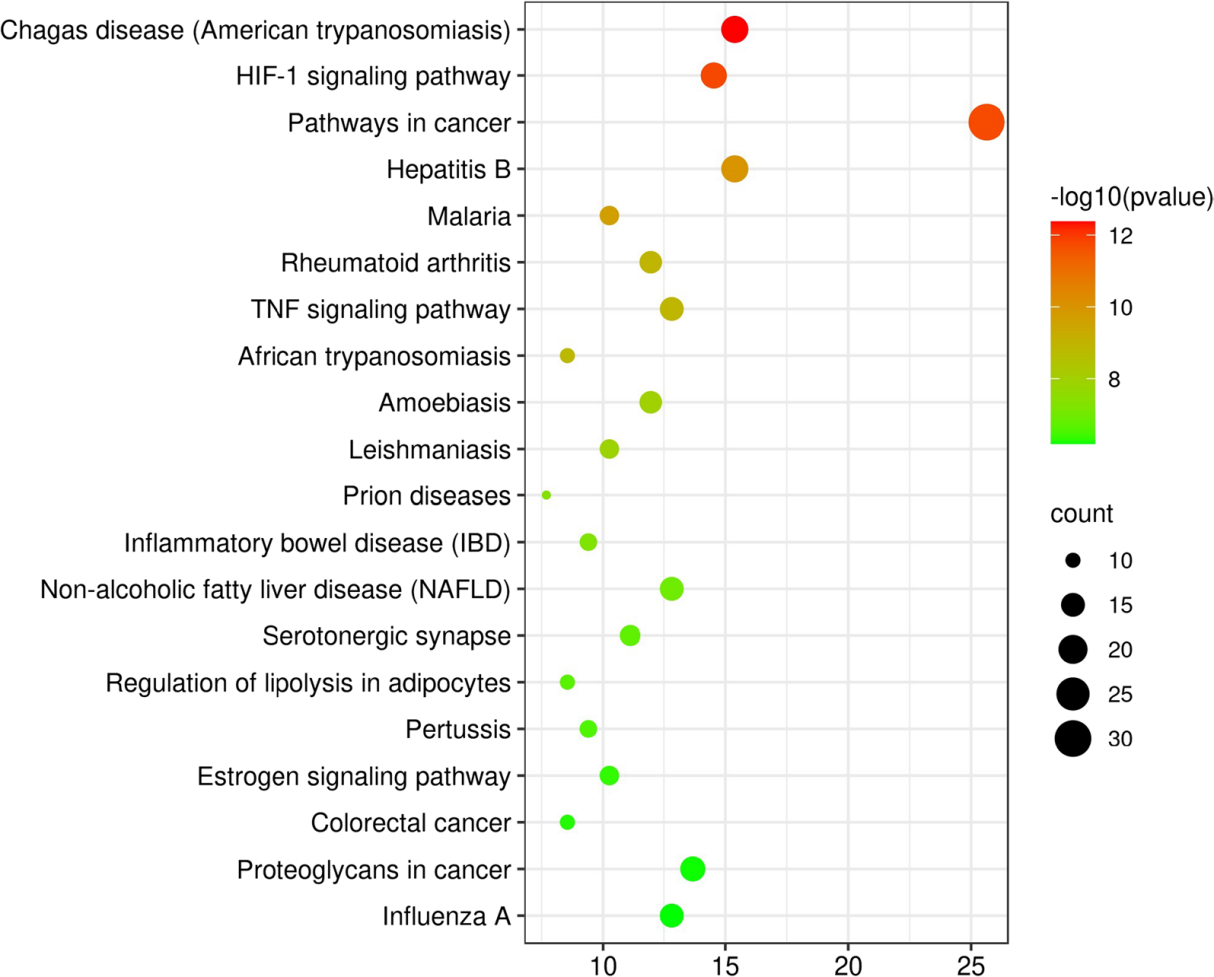
The top 20 signaling pathways in KEGG enrichment analysis of 117 intersection targets

We also conducted KEGG enrichment analysis through the DAVID database, and the results showed that these targets involved 124 pathways. We selected the top 20 according to the *p* value from small to large for further analysis (Fig. 8). It can be seen that JTW mainly regulates Chagas disease (American trypanosomiasis), HIF-1 signaling pathway, pathways in cancer, Hepatitis B and so on to treat DM and depression. In addition, there are also many targets enriched in pathways such as TNF signaling pathway, PI3K-Akt signaling pathway and MAPK signaling pathway, indicating that they may also play an important role in the treatment.

Afterwards, in order to explore the relationship between the top 20 pathways, drugs and intersection targets, we integrated the collected information and conducted a network by using the Cytoscape (Additional file 3: Fig. S2). It is obvious from the network that each pathway can correspond to multiple targets, and each target can also connect to multiple pathways. Different pathways can be connected to each other through the intersection targets, which fully reflects the multi-component, multi-target, and multi-pathway mechanism of JTW in treating DM and depression.

## Discussion

Both DM and depression are serious chronic diseases, which can lead to a serious decline in the quality of life, increase functional disability and costs of care than many other chronic diseases (O'Connor et al. 2009). The bidirectional link between DM and depression has been confirmed. An epidemiological study has shown that the prevalence rate of depression is more than three times higher in people with type 1 diabetes mellitus (T1DM) and nearly twice as high in people with T2DM than those without (Roy and Lloyd 2012). Another research also indicated that the presence of diabetes doubles the odds of comorbid depression (Anderson et al. 2001). The results of a meta-analysis carried out by Mezuk et al. showed that compared with people without diabetes, people with T2DM had a 15% increased risk of depression, while those with depression had a 60% increased risk of developing T2DM (Mezuk et al. 2008). However, the current treatments for these two diseases are relatively single and there is a lack of the comprehensive treatment needed to improve clinical outcomes (Lloyd et al. 2018).

JTW, one of the most classical Chinese prescription, has been used to treat insomnia for hundreds of years. In recent years, more and more experiments have confirmed that JTW can not only improve the quality of sleep, but also has the dual effects of hypoglycemic and antidepressant (Hu et al. 2013; Dong et al. 2013; Jiao et al. 2021). In the traditional theories of TCM, different diseases may have similar etiology, pathogenesis, symptoms, and disease location during the occurrence and development, so that different diseases can be cured with the same prescription, which fully reflects the advantages of TCM in syndrome differentiation, holistic treatment and comprehensive treatment (Yu et al. 2020). Our study is to evaluate the dual therapeutic effects of JTW, and to explore the potential mechanisms via network pharmacology.

The results of analysis using Cytoscape showed that the active compounds with the highest number of targets included quercetin, styrene, cinnamic acid, ethyl-cinnamate, (R)-Canadine, palmatine, berberine and so on. First of all, quercetin, one of the active compounds of HL, has been confirmed had multiple pharmacological effects (Duarte et al. 2001; Kumar et al. 2020; Sharma et al. 2018; D'Andrea 2015). Studies have found that quercetin could ameliorate metabolic derangements in diabetes and effectively improve dyslipidemia in T2DM (Roslan et al. 2017; Jeong et al. 2012). Not only that, quercetin has also been found to alleviate LPS-induced depression-like behaviors in rats (Fang et al. 2019), and can dose-dependently decrease the immobility time of diabetic mice in FST and this effect is comparable to that of fluoxetine, a traditional antidepressant (Anjaneyulu et al. 2003; Bhutada et al. 2010).

Berberine, also an active compound of HL, has a wide range of pharmacological effects (Wang et al. 2017; Fan et al. 2019). The results of a clinical trial showed that berberine can significantly reduce FBG and HbA1c in patients with diabetes, and its hypoglycemic effect was similar to that of metformin (Yin et al. 2008). The therapeutic effect of berberine on depression has also been confirmed by numerous experiments. Studies have found that berberine can exert antidepressant effect by inhibiting neuroinflammation and regulating brain biogenic amines (Yin et al. 2008; Hu et al. 2019). What’s more, berberine can greatly shorten the immobility time of mice in FST and TST in animal experiments (Peng et al. 2007; Lee et al. 2012).

Cinnamic acid, one of the effective compounds of RG, is a natural aromatic carboxylic acid. Research results show that cinnamic acid can regulate glycogen production and gluconeogenesis (Huang and Shen 2012), and can also significantly enhance insulin secretion in isolated islets (Hafizur et al. 2015), thereby exerting anti-diabetic activity. In addition, Hemmati et al. found that the administration of cinnamic acid can inhibit the FBG level in diabetic mice (Hemmati et al. 2018). Although there are few researches on the therapeutic effect of cinnamic acid in depression, derivatives of cinnamic acid and other natural products can exert antidepressant effects and have potential applicability as candidates for antidepressant drugs (Diniz et al. 2019).

Inflammatory mediators have always been considered to be important factors in promoting the development of insulin resistance (IR), which will lead to the occurrence of T2DM. The results of our PPI network analysis that IL-6 and TNF are the top five targets also confirmed this view, indicating that they may play an important role in the treatment of DM and depression. It was found that the levels of cytokines such as TNF-α and IL-6 were highest in non-treated diabetic rats, and decreased significantly following quercetin or glibenclamide treatments (Roslan et al. 2017). The systematic review conducted by Esser et al. directly showed that immune system activation and chronic low-grade inflammation are involved in the pathogenesis of IR and diabetes (Esser et al. 2014). Inflammation is also thought to have a bidirectional relationship with depression (Beurel et al. 2020). A meta-analysis demonstrated that there was a significant correlation between depression and C-reactive protein (CRP) and IL-6 in children and adolescents (Colasanto et al. 2020). IL-6 knockout mice exhibit resistance to stress-induced depression-like behavior and showed reduced despair in FST and TST (Chourbaji et al. 2006). Vascular endothelial growth factor (VEGF) is a key driver of neovascularization and vascular permeability (Abdelsaid and El-Remessy 2012; Kajdaniuk et al. 2011). A case–control study showed that altered VEGF secretion, caused by genetic variation in VEGF-A gene, is involved in T2DM pathogenesis (Sellami et al. 2018). In addition, VEGF exerts effective neurotrophic effects. In both major depressive disorder (MDD) subjects and rat depression models, the hippocampal VEGF and other growth factors are abnormally regulated (Carboni et al. 2018). Deyama et al. confirmed that VEGF signaling plays a crucial role in the antidepressant effects of brain-derived neurotrophic factor (BDNF) and ketamine (Deyama et al. 2019a, b). It can be seen that VEGF is closely related to DM and depression. The correlation between INS and DM has long been recognized worldwide, and AKT1, as a key factor in the PI3K-Akt signaling pathway, has been confirmed by many researches on its relationship with DM and depression, which will be discussed in detail below.

The results of KEGG enrichment analysis showed that the active compounds of JTW may play a therapeutic effect on DM and depression by regulating multiple pathways. The intersection targets are mainly enriched in HIF-1 signaling pathway, pathways in cancer, TNF signaling pathway, PI3K-Akt signaling pathway and MAPK signaling pathway, etc. Many studies have shown that HIF-1 signaling pathway is associated with DM and depression. HIF-1α is important for maintaining the function and survival of pancreatic β cells (Stokes et al. 2013) and the expression of HIF-1β mRNA in patients with T2DM is decreased (Gunton et al. 2005). Glucose-induced inhibition of HIF-1α protein stability may also accelerate the deterioration of β cell function and speed progression to diabetes (Cheng et al. 2010). In terms of depression, Li et al. established a depression model using chronic unpredictable mild stress (CUMS) and found that FG-4592 can reverse depressive behaviors by activating HIF-1 signaling pathway (Li et al. 2020). Kang et al. also proposed that interventions including the intermittent hypoxia conditioning and hyperbaric oxygen therapy to elevate the level of HIF-1 in the brain might be considered as new additional treatments for depression (Kang et al. 2021).

PI3K-Akt signaling pathway is the main downstream molecular pathway of insulin, which plays a crucial role in regulating glucose and lipid metabolism. PI3K-Akt signaling pathway block and abnormal function of downstream target proteins can cause IR (Bathina and Das 2018). Lots of studies have confirmed that the IR of diabetic mice can be improved by regulating the PI3K-Akt signaling pathway (Chen et al. 2019; Yan et al. 2018; Liao et al. 2019). AKT1 has been considered as a key mediator of insulin-stimulated glucose uptake, suppression of apoptosis, stimulation of glycolysis and the activation of glycogen and protein synthesis (Coffer et al. 1998). The activation of PI3K-Akt pathway can protect pancreatic β cells from the influence of different apoptotic stimuli (Tuttle et al. 2001). What’s more, Cao et al. found that the expression of PI3K in depressed rats were attenuated significantly (Cao et al. 2019). The study of Xie et al. showed that Crocin can ameliorate depression via PI3K-Akt mediated suppression of inflammation (Xie et al. 2019), these studies illustrate the close connection between PI3K-Akt signaling pathway and depression.

In mammalian cells, MAPK families has been divided into three categories, including p38, extracellular signal-related kinase (ERK) and c-Jun N-terminal kinase (JNK) (Dewanjee et al. 2018). Among them, ERK1/2 play a pivotal role in various neuropsychiatric disorders, including depression (Wang and Mao 2019). Regulating the CUMS-induced MAPK pathway and NF-κB protein complex activation can alleviate depression-like behavior in mice (Su et al. 2017). Paroxetine combined with fluorouracil, ketamine and ghrelin and other drugs can show antidepressant-like effects via the MAPK signaling pathway (Zhang et al. 2020; Humo et al. 2020; Han et al. 2019). Cui et al. found that HL can alleviate inflammation by regulating the expression of pro-inflammatory cytokines through MAPK signaling pathway, thereby inhibiting the occurrence and development of IR and diabetes (Cui et al. 2018). *Gelidium elegans* extract can ameliorate T2DM via regulation of MAPK and PI3K-Akt signaling pathways (Choi et al. 2018).

As expected in our study, JTW has a therapeutic effect on diabetic mice, and can ameliorate depression-like behaviors induced by CRS. We found that JTW reduced the serum IL-6, TNF-α and hs-CRP levels, suggesting that JTW could by modulating the inflammation-related pathways, which were also predicted in the follow-up study by network pharmacological approach.

In our animal experiment, CRS was used to establish a DM combined with depression mice model in db/db mice, after conducting the behavioral tests, detecting serum inflammatory biomarkers and depression-related neurotransmitters, and observing apoptotic cells in the hippocampus of each mouse in different groups, we found that JTW did have such dual effects in treating DM and depression. Meanwhile, we explored the potential mechanism through network pharmacology. However, due to the limitations of some databases, we cannot collect all the active compounds and targets of JTW, and the targets and pathways are interrelated and regulate each other, more in-depth and comprehensive study is still needed, and in vivo and in vitro experiments are necessary for exploring and verifying more extensive pharmacological effects and mechanisms of JTW.

## Conclusion

Our findings suggested that JTW has a therapeutic effect on diabetic mice, and can ameliorate depression-like behaviors induced by CRS, that is to say, JTW has dual effects on DM and depression. Network pharmacology analysis revealed the multi-component, multi-target, and multi-pathway mechanism of JTW. The key targets of JTW in treating DM and depression probably were INS, AKT1, IL-6, VEGF-A and TNF, and the underlying mechanism would be associated with modulation on HIF-1 signal pathway, pathways in cancer, Hepatitis B, TNF signal pathway, PI3K-Akt signal pathway and MAPK signaling pathway and so on. Our study provides evidence for JTW in DM and depression therapy, and would provide a theoretical basis for the experimental research and clinical application of JTW in the future.

## Supplementary Information

**Additional file 1: Table S1.** Twenty-eight potential active compounds of Jiao-tai-wan and their corresponding OB and DL.

**Additional file 2: Figure S1.** The top 20 BP, MF and CC in GO analysis of 117 intersection targets.

**Additional file 3: Figure S2.** Drug-intersection targets-signaling pathways network of Jiao-tai-wan in the treatment of diabetes mellitus and depression.

## Data Availability

The raw data supporting the conclusion of this article will be made available by the authors, without undue reservation, to any qualified researcher.
