# Supplementary material for: The effect and mechanism of Jiao-tai-wan in the treatment of diabetes mellitus with depression based on network pharmacology and experimental analysis
Source: Mol Med. 2021 Dec 7;27:154. doi: 10.1186/s10020-021-00414-z (PMC8650382; doi:10.1186/s10020-021-00414-z)
Supplement: Supplementary file 1 — Additional file 1: Table S1. Twenty-eight potential active compounds of Jiao-tai-wan and their corresponding OB and DL. [file 10020_2021_414_MOESM1_ESM.docx]

Additional Table 1. Twenty-eight potential active compounds of Jiao-tai-wan and their corresponding OB and DL.

| Mol ID | Molecule Name | OB (%) | DL |
| --- | --- | --- | --- |
| MOL000004 | [Procyanidin B1](http://www.megabionet.org/tcmid/ingredient/17862/) | 67.87 | 0.66 |
| MOL000098 | quercetin | 46.43 | 0.28 |
| MOL000105 | [Protocatechuic Acid](http://www.megabionet.org/tcmid/ingredient/23246/) | 25.37 | 0.04 |
| MOL000431 | [Coumarin](http://www.megabionet.org/tcmid/ingredient/4140/) | 29.17 | 0.04 |
| MOL000475 | [Anethole](http://www.megabionet.org/tcmid/ingredient/1186/) | 32.49 | 0.03 |
| MOL000622 | Magnograndiolide | 63.71 | 0.19 |
| MOL000704 | [Styrene](http://www.megabionet.org/tcmid/ingredient/20414/) | 29.55 | 0.01 |
| MOL000762 | Palmidin A | 35.36 | 0.65 |
| MOL000785 | palmatine | 64.6 | 0.65 |
| MOL000991 | [Cinnamaldehyde](http://www.megabionet.org/tcmid/ingredient/3693/) | 31.99 | 0.02 |
| MOL001454 | berberine | 36.86 | 0.78 |
| MOL001458 | coptisine | 30.67 | 0.86 |
| MOL002225 | [Cinnamic Alcohol](http://www.megabionet.org/tcmid/ingredient/30783/) | 38.35 | 0.02 |
| MOL002295 | [Cinnamic Acid](http://www.megabionet.org/tcmid/ingredient/23095/) | 19.68 | 0.03 |
| MOL002668 | Worenine | 45.83 | 0.87 |
| MOL002834 | [Ethylcinnamate](http://www.megabionet.org/tcmid/ingredient/7429/) | 20.54 | 0.04 |
| MOL002894 | berberrubine | 35.74 | 0.73 |
| MOL002897 | epiberberine | 43.09 | 0.78 |
| MOL002903 | (R)-Canadine | 55.37 | 0.77 |
| MOL002904 | Berlambine | 36.68 | 0.82 |
| MOL002907 | Corchoroside A_qt | 104.95 | 0.78 |
| MOL003526 | [Cinnamyl Acetate](http://www.megabionet.org/tcmid/ingredient/3726/) | 21.15 | 0.04 |
| MOL003530 | O-Methoxycinnamaldeh | 26.52 | 0.04 |
| MOL007283 | [Procyanidin B2](http://www.megabionet.org/tcmid/ingredient/17862/) | 3.01 | 0.66 |
| MOL007285 | [Procyanidin C1](http://www.megabionet.org/tcmid/ingredient/17879/) | 18.98 | 0.1 |
| MOL008647 | Moupinamide | 86.71 | 0.26 |
| MOL009089 | [Procyanidin B5](http://www.megabionet.org/tcmid/ingredient/17874/) | 3.01 | 0.73 |
| MOL013352 | Obacunone | 43.29 | 0.77 |
