# Supplementary figures and images for: The effect and mechanism of Jiao-tai-wan in the treatment of diabetes mellitus with depression based on network pharmacology and experimental analysis
Source: Mol Med. 2021 Dec 7;27:154. doi: 10.1186/s10020-021-00414-z (PMC8650382; doi:10.1186/s10020-021-00414-z)

A

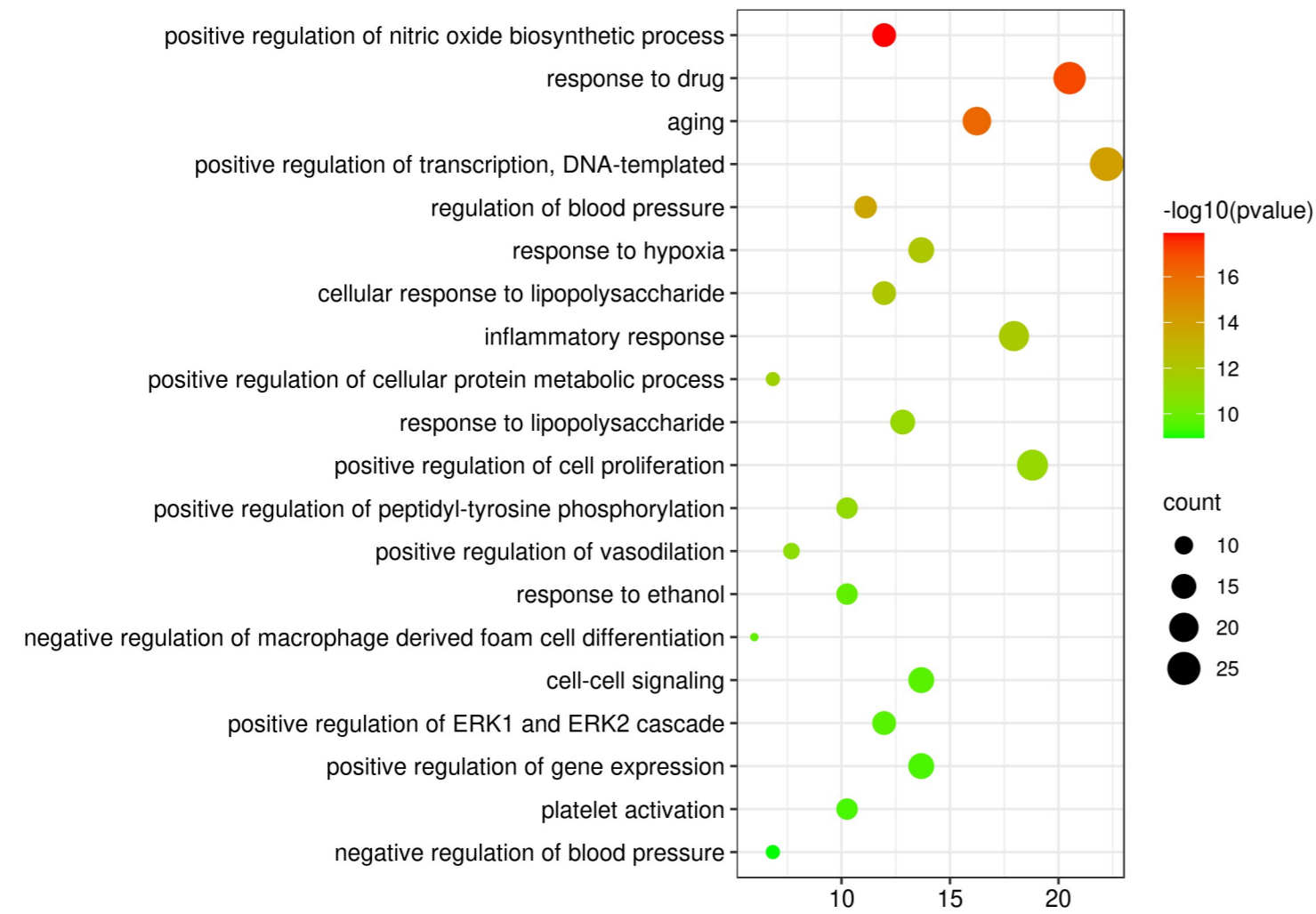

B

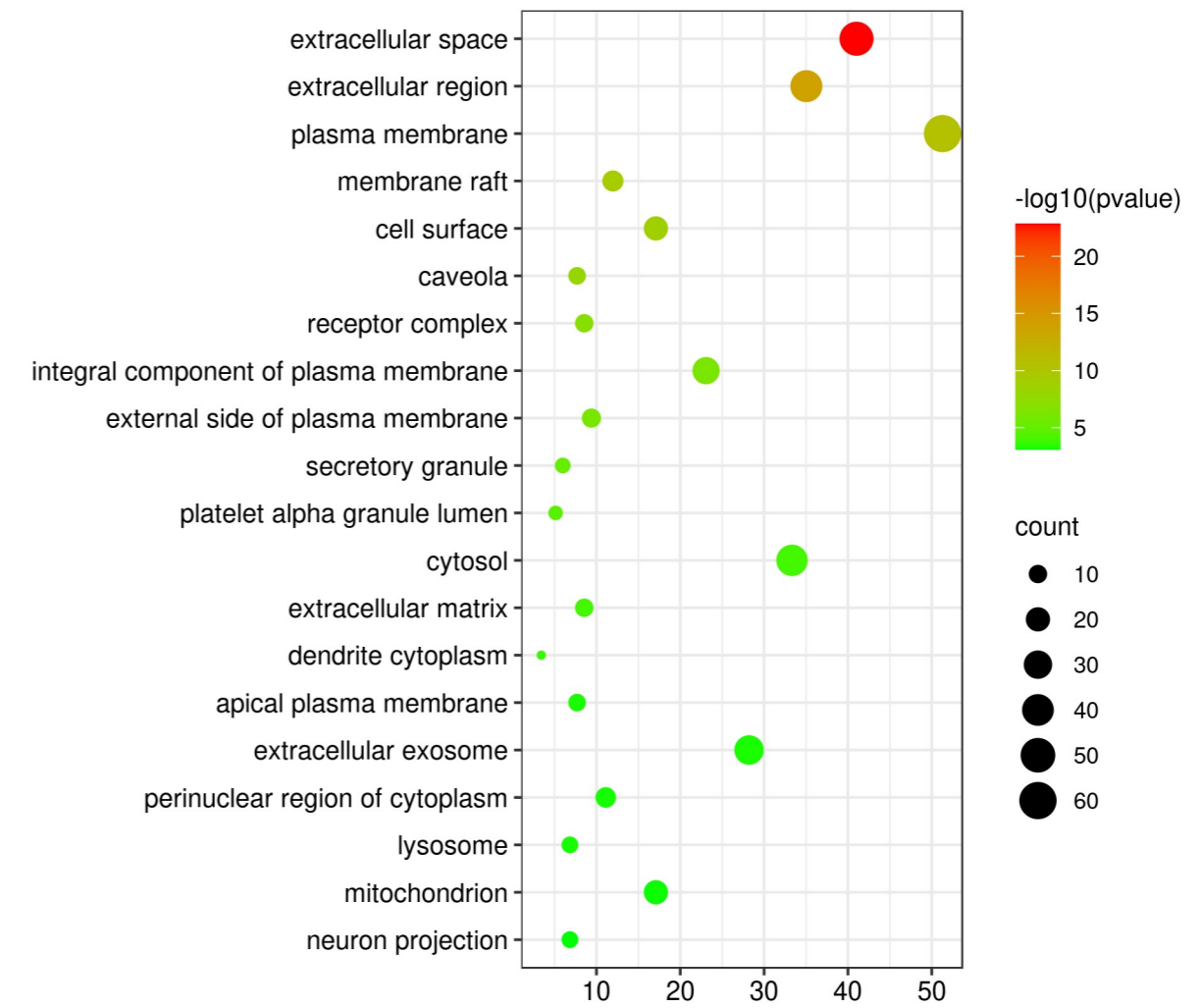

C

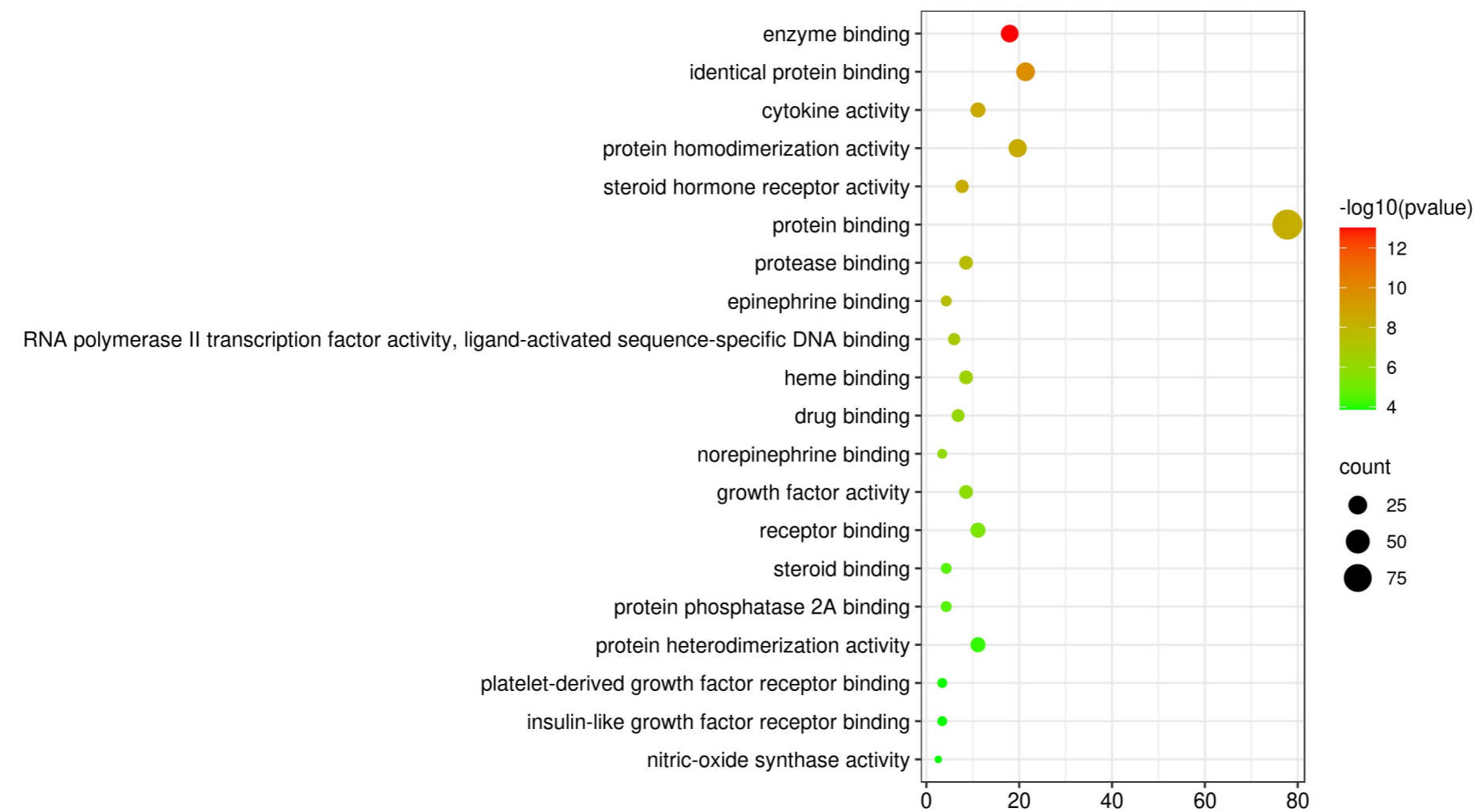

Supplement: Supplementary file 2 — Additional file 2: Figure S1. The top 20 BP, MF and CC in GO analysis of 117 intersection targets. [file 10020_2021_414_MOESM2_ESM.pdf]

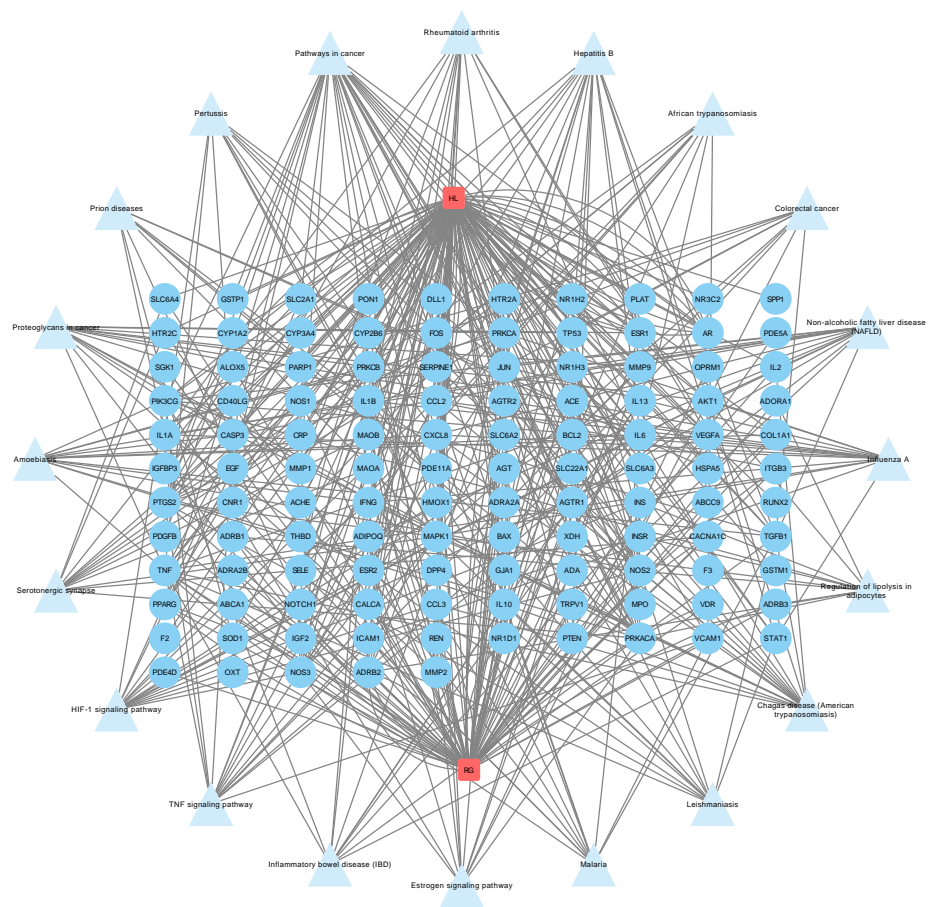

Supplement: Supplementary file 3 — Additional file 3: Figure S2. Drug-intersection targets-signaling pathways network of Jiao-tai-wan in the treatment of diabetes mellitus and depression. [file 10020_2021_414_MOESM3_ESM.pdf]
